# CXCL12/CXCR4 axis as a key mediator in atrial fibrillation via bioinformatics analysis and functional identification

**DOI:** 10.1038/s41419-021-04109-5

**Published:** 2021-08-27

**Authors:** Peng Liu, Hongke Sun, Xin Zhou, Qiaozhu Wang, Feng Gao, Yuping Fu, Tong Li, Yixin Wang, Yingqi Li, Boyuan Fan, Xiaoli Li, Tiannan Jiang, Xinghua Qin, Qiangsun Zheng

**Affiliations:** 1 Department of Cardiology, The Second Affiliate Hospital of Xi’an Jiaotong University, Xi’an, China; 2 Department of Cardiology, The First Affiliate Hospital of Xi’an Jiaotong University, Xi’an, China; 3 School of Life Sciences, Northwestern Polytechnical University, Xi’an, China; 4 Department of Internal Medicine, Health Care Center, Beijing Friendship Hospital, Capital Medical University, Beijing, China

**Keywords:** Gene expression profiling, Chemokines, Molecular biology, Atrial fibrillation

## Abstract

Atrial fibrillation (AF) is an increasingly prevalent arrhythmia with significant health and socioeconomic impact. The underlying mechanism of AF is still not well understood. In this study, we sought to identify hub genes involved in AF, and explored their functions and underlying mechanisms based on bioinformatics analysis. Five microarray datasets in GEO were used to identify the differentially expressed genes (DEGs) by Robust Rank Aggregation (RRA), and hub genes were screened out using protein–protein interaction (PPI) network. AF model was established using a mixture of acetylcholine and calcium chloride (Ach-CaCl_2_) by tail vein injection. We totally got 35 robust DEGs that mainly involve in extracellular matrix formation, leukocyte transendothelial migration, and chemokine signaling pathway. Among these DEGs, we identified three hub genes involved in AF, of which CXCL12/CXCR4 axis significantly upregulated in AF patients stands out as one of the most potent targets for AF prevention, and its effect on AF pathogenesis and underlying mechanisms were investigated in vivo subsequently with the specific CXCR4 antagonist AMD3100 (6 mg/kg). Our results demonstrated an elevated transcription and translation of CXCL12/CXCR4 axis in AF patients and mice, accompanied with the anabatic atrial inflammation and fibrosis, thereby providing the substrate for AF maintenance. Blocking its signaling via AMD3100 administration in AF model mice reduced AF inducibility and duration, partly ascribed to decreased atrial inflammation and structural remodeling. Mechanistically, these effects were achieved by reducing the recruitment of CD3+ T lymphocytes and F4/80+ macrophages, and suppressing the hyperactivation of ERK1/2 and AKT/mTOR signaling in atria of AF model mice. In conclusion, this study provides new evidence that antagonizing CXCR4 prevents the development of AF, and suggests that CXCL12/CXCR4 axis may be a potential therapeutic target for AF.

## Introduction

Atrial fibrillation (AF) is a highly prevalent cardiac arrhythmia with significant health and socioeconomic impact [1]. Apart from worsening patient quality of life, AF is associated with stroke, new-onset heart failure, dementia, and increased mortality [2]. Despite significant effort has been done to clarify the molecular and cellular mechanisms underlying AF, the advancement is obstructed by the reality that AF is a complex arrhythmia. New drugs specially designed for the therapy of AF remain suboptimal and patients, especially with persistent AF, have to depend on antique antiarrhythmic drugs, such as amiodarone, sotalol, propafenone, and flecainide that have limited efficacy and significant side effects [3]. Improving the understanding of AF mechanisms and identifying regulatory pathways are pivotal to prevent its perpetuation. In date, numerous researches have suggested that AF cases in the general population have a significant genetic component, even beyond traditional risk factors [4, 5]. Therefore, exploring transcriptome data with bioinformatics analysis may be detect some potential mechanisms of AF to provide novel mechanism-targeting therapies and aid in future clinical studies [6].

CXCL12 (C-X-C chemokine ligand 12), also known as stromal-derived factor 1 (SDF-1), is a small proinflammatory chemoattractant cytokine [7–11]. The CXCR4 (C-X-C chemokine receptor type 4) as the receptor of CXCL12 belongs to the family of seven-span transmembrane G-protein-coupled chemokine receptors (GPCRs) [7–9]. The CXCL12/CXCR4 axis participates in complex biological processes, such as homeostatic immune cell migration, inflammatory response, tissue repair, and cell survival, which has also been considered as an attractive therapeutic target for various cardiac and noncardiac diseases [9, 10, 12]. Previous clinical data have implicated that upregulated expression of CXCL12 and CXCR4 in plasma or atria of AF patients is associated with excessive atrial remodeling, longer hospital stay, and even a higher mortality risk [13–15]. Nevertheless, the precise mechanism for CXCL12/CXCR4 axis in AF has not been fully understood.

**Fig. 1:**
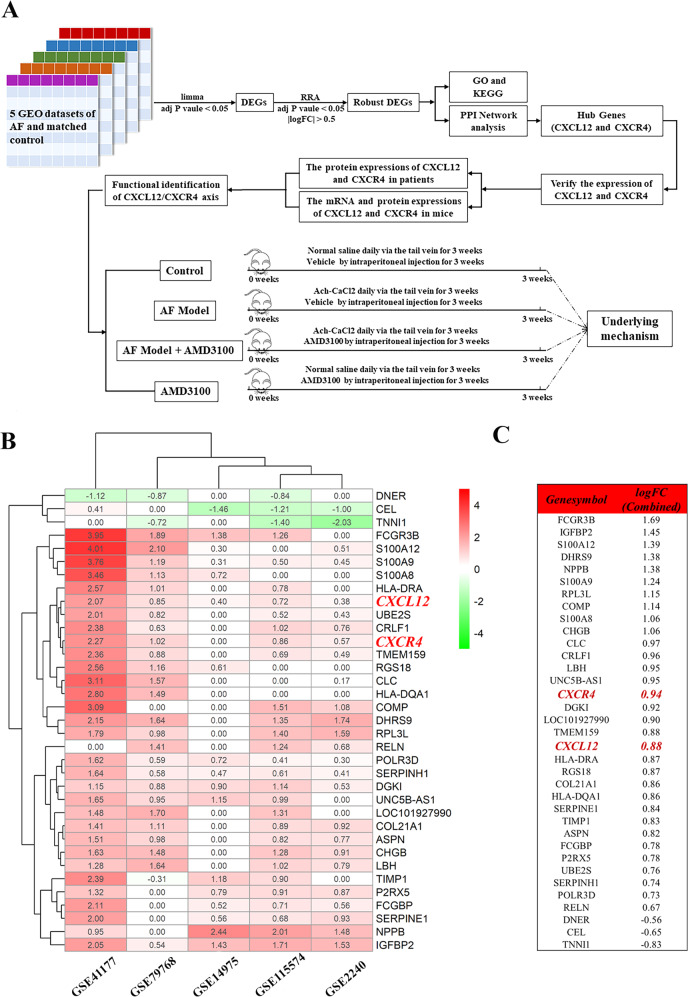
The research route and DEGs between AF and matched control by RRA analysis. **A** The research route and framework. **B** Heatmap shows the 35 DEGs between the AF group and matched control. There are 32 upregulated genes and three downregulated genes in AF group compared with the control group. Each row represents one gene and each column indicates one dataset. The red rectangles indicate the genes are upregulated in AF group, on the contrary, the green rectangles indicate the genes are downregulated in AF group. The number in the rectangle is the log2 FC of the gene (compare to the control group) in 5 respective datasets. **C** The combined log2 FC of 35 DEGs calculated by RRA based on 5 datasets. The combined log2 FC of CXCR4 and CXCL12 are 0.94 and 0.88, respectively, which means these two genes are significantly upregulated in AF group. DEG differentially expressed gene, GEO gene expression omnibus, RRA robust rank aggregation, GSE gene expression omnibus series, log2 FC log2 fold change.

In this study, we applied bioinformatics tools to analyze five Gene Expression Omnibus (GEO, http://www.ncbi.nlm.nih.gov/geo/) datasets with the goal of identifying differentially expressed genes (DEGs) between AF and matched controls. The results of bioinformatics analysis suggest that CXCL12/CXCR4 axis may function pivotally in pathological processes of AF. Therefore, we aim to further verify the expression profile of CXCL12/CXCR4 axis in AF patients and mice and elucidate its role and underlying mechanisms in AF development, in order to provide a novel target for optimizing the treatment of AF (Fig. 1A).

## Materials and methods

### Retrieve the microarray gene expression datasets of AF in GEO

The microarray gene expression datasets about AF and matched control were retrieved from the GEO Datasets. We take “atrial fibrillation” as the keyword for the retrieval in GEO Datasets. The “Homo sapiens” and “Expression profiling by array” were as filter conditions for “Organism” and “Study type,” respectively. The selection criteria for microarray datasets of AF were as follows: (1) tissue samples of atria or appendage from patients with AF and sinus rhythm control group, (2) gene expression profiling of mRNA, and (3) sample count of each group are at least three. According to the above screening criteria, five AF microarray gene expression datasets were included in this study: GSE2240 (ref [16].), GSE14975 (ref [17].), GSE41177 (ref [18].), GSE79768 (ref [19].) and GSE115574 (no citation, https://www.ncbi.nlm.nih.gov/geo/query/acc.cgi.). These five microarray gene expression databases contained 53 AF patients and 49 sinus rhythm controls. The detailed information of these five GEO datasets were listed in (Supplementary file 1).

### Microarray data processing and DEGs’ identification

All of the raw data (CEL file) and probe annotation files of microarrays were downloaded from GEO. R packages of “affy” and “affyPLM” provided by a Bioconductor project were used to assess chip quality [20]. The raw data were preprocessed via background correction, quantile normalization, and calculating expression using the robust multi-array average (RMA) algorithm [21]. Gene expression fold changes (FC) were computed by subtracting the mean of normalized log2-based expression levels of the respective control groups from each subject’s normalized log2-based expression levels. The R package “limma” was utilized to find DEGs between AF and matched control in each dataset [22]. The Benjamini‑Hochberg (BH) method was used to adjust original p-values and adjusted *P* < 0.05 was used to filter DEGs. Subsequently, the Robust Rank Aggregation (RRA) was applied to rank the DEGs of these five datasets to find robust DEGs by criteria of adjust *P* < 0.05 and genes expression values of the | log2 FC | >0.5 (ref [23].).

### Functional enrichment analysis of DEGs

We conducted gene ontology (GO) and Kyoto Encyclopedia of Genes and Genomes (KEGG) pathway enrichment analyses to predict the potential function of robust DEGs by the R package “Clusterprofiler” [24]. GO terms and KEGG pathways with adjust *P* < 0.05 were considered statistically significant.

### Protein–protein interaction (PPI) network of DEGs and hub genes’ identification

PPI network of robust DEGs was constructed by the search tool for the retrieval of interacting genes (STRING database, V11.0; http://string-db.org/) to predict protein functional associations. Subsequently, the network was visualized by Cytoscape software (V3.8.0; http://cytoscape.org/) [25]. Then, we assessed the “degree” of every node in the interaction network by a Cityscape’s plugin CytoHubba [26]. In this network, a node represents a protein (gene), and lines represent interactions of the proteins. The “degree” of each node is equal to the number of nodes interacting with this node. The higher the degree is, the closer the connections with other nodes are, indicating a higher importance of the node in the network. The top three genes, as ranked by degree, were considered the hub genes of AF, which would be further researched in vivo.

### Human atrial tissues and ethical statement

To confirm the expression of CXCL12 and CXCR4 in AF patients and healthy controls, 10 AF patients and seven matched controls (undergoing open-heart surgery for valve repair or coronary artery bypass grafting) of atrial tissues were obtained from the Second Affiliated Hospital of Xi’an Jiaotong University (Xi’an, China). Every specimen was anonymously handled based on ethical standards. The protocol was approved by the Ethics Committee of the Second Affiliated Hospital of Xi’an Jiaotong University and written informed consent was obtained from all patients. Clinical characteristics of patients enrolled in this study are presented in (Supplementary file 2). The protein expressions of CXCL12 and CXCR4 in paraffin-embedded samples from 17 patients were detected by immunohistochemical staining.

### Animal model and experimental procedures

A total of 60 C57BL/6 J male mice (8-week-old) were obtained from the Experimental Animal Center of Medical School, Xi’an Jiaotong University. AF model was established by a mixture of Ach (66 μg/kg; Shanghai Macklin Biochemical Co., Ltd., Shanghai, China) and CaCl_2_ (10 mg/kg; Shanghai Macklin Biochemical Co., Ltd., Shanghai, China) by tail vein injection (i.v.) for 3 weeks [27–29]. The mice were concurrently treated with specific CXCR4 antagonist AMD3100 (ref [30].) (6 mg/kg; Topscience, Shanghai, China) or vehicle (normal saline) by intraperitoneal injections (i.p.) for 3 weeks [31, 32]. Specifically, the study included the following treatment groups: control (n = 15), AF model (Ach-CaCl_2_; n = 15), AF model + AMD3100 (Ach-CaCl_2 _+ AMD3100; *n* = 15), and AMD3100 alone (*n* = 15). 3 weeks later, echocardiography and electrophysiological examination were performed before tissue sampling. The animal study was approved by the Institutional Ethics Committee for Animal Experiments of Xi’an Jiaotong University. All procedures conformed to the Guide for the Care and Use of Laboratory Animals.

### Transthoracic echocardiography

Transthoracic echocardiography was performed with the Vevo 1100 imaging (VisualSonics, Toronto, Canada) to detect cardiac structural and functional indicators. The parameters, such as left atrium diameter (LAD), interventricular septal thickness at end-systole (IVSd); interventricular septal thickness at end-systolic (IVSs); left ventricular ejection fractions (LVEF), left ventricular fractional shortening (LVFS), left ventricular internal diameter at end-diastole (LVIDs), left ventricular internal diameter at end-systole (LVIDd), left ventricular posterior wall thickness at end-diastole (LVPWd), left ventricular posterior wall thickness at end-systole (LVPWs) were measured. Furthermore, peak velocities of the early (MV E) and late (MV A) phases of the mitral inflow from the Doppler recordings were measured and their ratio (E/A) calculated. Echocardiography was performed by a technician who was blind to the grouping of the mice. Every parameter was measured three times and the average values were calculated.

### Electrophysiological analysis

Electrophysiological analysis was performed by surface electrocardiogram (ECG) and intraesophageal burst pacing. After mice were anesthetized with sodium pentobarbital (50 mg/kg, i.p.). The electrode needles were inserted subcutaneously into the fixed mice limbs. Surface ECGs were recorded and amplified by RM6240E physiological signal acquisition system (Chengdu Instrument Factory, Chengdu, China). The ECG parameters, such as P-wave duration, PR interval, QRS duration, and QT interval were measured [33]. The intraesophageal burst pacing was performed as previously described with VCS-3001 stimulator (MappingLab, Oxford, UK) [33, 34]. Briefly, a 2.0 French 4-polar electrodes catheter (interelectrode distance 2.0 mm) was inserted into the esophagus and positioned near the left atrium. Regular pacing and standard S1S2 pacing protocols were used to determine the standard electrophysiological parameters, such as sinus node recovery time (SNRT), sinoatrial conduction time (SACT), and atrioventricular nodal refractory period (AVERP). To assess AF susceptibility, burst pacing was applied at three different frequencies: 30 Hz, 35HZ, and 40 Hz (3-s burst pacing, 5-s intervals, total 10 continuously bursts pacing in each frequency) with two times the threshold current, respectively. The successful induction of AF was defined as a period of rapid irregular atrial rhythm for at least 1 s. The total time of AF episodes was defined as the sum of the AF duration of each episode.

### Atrial histology analysis

Animals were killed at the end of the experiments by deep anesthesia followed by the rapid removal of the heart. The hearts were weighed, and either fixed in 4% formalin in PBS for sectioning or snap-frozen in liquid nitrogen for molecular biology. Four-micron paraffin sections of atria were stained with Masson’s trichrome to evaluate the distribution and localization of collagen. The extent of interstitial fibrosis was quantified in each of three random fields per section, using ImageJ software (Bethesda, Maryland, USA) coupled to a TS100 microscope (Nikon, Tokyo, Japan) at a magnification of X 400.

The immunohistochemistry (IHC) analysis was performed on paraffin-embedded samples of atria of patients and mice. Paraffin blocks were sectioned to 4 µm thickness. The sections were dewaxed, rehydrated, and antigen-repaired. Then, the primary antibodies, including CXCL12 antibody (1:200; Servicebio, Wuhan, China), CXCR4 antibody (1:1000; Servicebio), rabbit-antimouse CD3 antibody (1:100; Servicebio), rabbit-antimouse F4/80 antibody (1:800; Servicebio), and rabbit-antimouse CD20 antibody (1:800; Servicebio) were added and incubated overnight at 4 °C for identification of CXCL12, CXCR4, CD3+ T lymphocytes, CD20+ B lymphocytes and F4/80+ macrophages. After washing three times in PBS, sections were incubated with the appropriate biotinylated secondary antibody (Servicebio) for 50 min at room temperature, and followed by incubation with DAB color developing solution (Servicebio). Subsequently, the sections are counterstained with hematoxylin stain solution and dehydrated. The nucleus of hematoxylin stained is blue, and the positive expression of DAB is brownish yellow. Images were captured under TS100 microscope (Nikon) at a magnification of X 400, and then analyzed using ImageJ software (Bethesda).

### Immunoblotting analysis

Protein lysates were extracted from atria, and the concentration was determined using a bicinchoninic acid (BCA) protein assay. Proteins (20–40 μg) were subjected to sodium dodecyl sulfate-polyacrylamide gel electrophoresis and transferred to a polyvinylidene difluoride membrane, which was incubated with primary antibodies against collagen-1 (1:1000; Abcam, Cambridge, UK), collagen-3 (1:1000; Abcam), α-SMA (1:3000; Abcam), MMP9 (1:3000; Abcam), p-AKT (1:1000; Cell Signaling Technology, Danvers, MA, USA), AKT (1:3000; Cell Signaling Technology), p-mTOR (1:3000; Cell Signaling Technology), mTOR (1:3000; Cell Signaling Technology), p-ERK1/2 (1:3000; Cell Signaling Technology), ERK1/2 (1:3000; Cell Signaling Technology) and GAPDH (1:3000; Cell Signaling Technology). The membranes were then incubated with antimouse or anti-rabbit IgG secondary antibodies (1:5000; Cell Signaling Technology) at room temperature for 1.5 h. Bands were visualized using a Tanon 4800 chemiluminescence detection system (Tanon, Shanghai, China). All blots were analyzed by using the ImageJ (Bethesda) and normalized to GAPDH levels.

### Quantitative real-time PCR (qPCR) analysis

Total RNA was isolated from fresh atrial tissue using TRIzol (AG, Changsha, China) according to the manufacturer’s protocol. The single-stranded cDNA was transcribed with the help of *Evo M-MLV* RT Kit (AG). The quantitative real-time polymerase chain reaction was used to detect gene levels of CXCL12, CXCR4, collagen-1, collagen-3, α-SMA, IL-1β, IL-6, IL-18, TNF-α, and β-actin in cardiac tissues using SYBR^®^ Green Premix Pro Taq HS qPCR Kit (AG) with RT-PCR Detection System (Bio-Rad, Hercules, CA) [35]. The PCR primers are listed in (Supplementary file 3). Gene expression was normalized to a reference gene (β-actin). The relative quantification of gene expression was determined with the 2^−ΔΔCT^ method [36].

### Statistical analysis

Statistical analysis was performed using SPSS version 25.0 software (SPSS Inc., Chicago, IL, USA). The data are presented as mean ± standard error of the mean (SEM). Normally distributed variables were compared by Student’s *t* test, or one-way analysis of variance (ANOVA) followed by Bonferroni correction for multiple comparisons. Chi-square test was used to analyze the counting data. *P* < 0.05 was considered statistically significant.

## Results

### The DEGs between AF patients and matched controls from the reanalysis of five GEO datasets

**Fig. 2:**
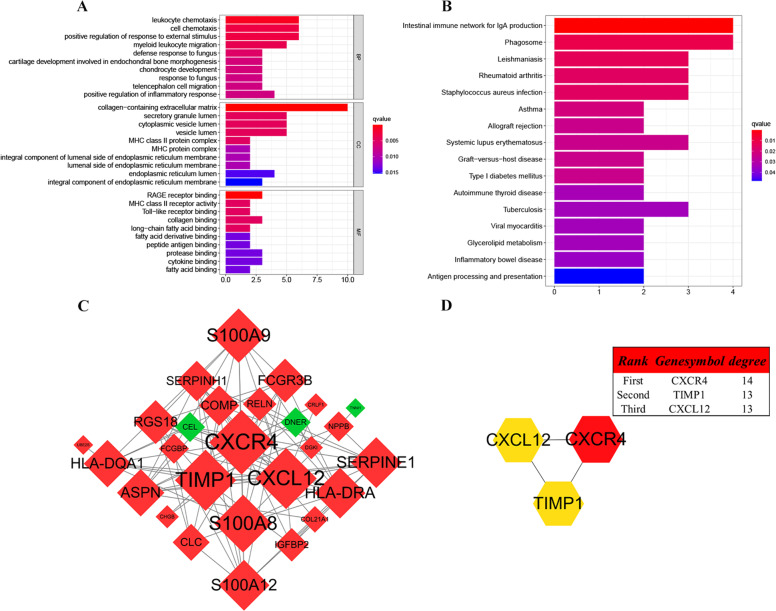
The DEGs’ GO and KEGG functional enrichment and PPI network. **A** GO functional enrichment of DEGs, which includes BP, CC, and MF. **B** KEGG pathway enrichment of DEGs. GO terms and KEGG pathways are presented in the bar chart. The *y-*axis depicts names of GO terms and KEGG pathways, and the *x*-axis depicts the count of genes in each GO term or KEGG pathway. The higher *q* value is shown in color blue, while the lower value is shown in red. **C** PPI network of DEGs. The nodes represent proteins, the edges represent the interaction of proteins. The green and red diamonds indicate downregulated and upregulated DEGs in AF group respectively. The size of the diamond is positively correlated with nodes’ degree calculated by Cytohubba in the network. The larger the diamond, the higher the degree of the protein represented by the node in the network. **D** The top three hub genes screened by degree in PPI network. Red, greater degree. Yellow, lesser degree. GO Gene Ontology, KEGG Kyoto Encyclopedia of Genes and Genomes, DEG differentially expressed gene, BP biological process, MF molecular function, CC cellular component, PPI protein–protein interaction.

We identified 6686, 20079, 5639, 15636, and 14178 DEGs in GSE2240, GSE41177, GSE14975, GSE79768, and GSE115574, respectively (Supplementary file 4). These DEGs were excavated in the subsequent RRA analysis. Finally, we got 35 robust DEGs, including 32 upregulated and three downregulated in AF (Fig. 1B, C, Supplementary file 5). Among these DEGs, CXCL12 and CXCR4 were significantly upregulated in the atria of AF patients compared with controls (Fig. 1B, C). Furthermore, GO and KEGG-enrichment analysis was performed to determine the biological features of these robust DEGs. GO function-enrichment analysis resulted in 134 items, of which biological processes (BP) accounted for 97, cell components (CC) for 18, and molecular function (MF) for 19 items (Supplementary file 6A). BP analysis reveals that these genes are markedly enriched for leukocyte chemotaxis and cell chemotaxis (Fig. 2A). CC analysis shows that these genes are mainly enriched in the collagen-containing extracellular matrix and secretory granule lumen (Fig. 2A). Alterations in the MF of these genes are significantly enriched in collagen binding and cytokine binding (Fig. 2A). Meanwhile, KEGG pathway analysis returned 16 items (Supplementary file 6B), mainly, including intestinal immune network for IgA production, phagosome, and chemokine signaling pathway (Fig. 2B).

### CXCL12 and CXCR4 are identified as hub genes which might be closely related with AF

The PPI network of robust DEGs were analyzed in the String database, followed by network visualization with Cytoscape software. The network consists of 27 nodes and 92 edges (Fig. 2C). Then, we screed out the top three hub genes by CytoHubba in the PPI network, which were CXCR4 (degree = 14), CXCL12 (degree = 13), and TIMP1 (degree = 13) (Fig. 2D). Among these three hub genes, we selected CXCL12 and CXCR4, which were rarely reported in AF before, to validate their function and molecular mechanisms underlying AF.

### The verification of the expression of CXCL12 and CXCR4 in AF patients and mice

**Fig. 3:**
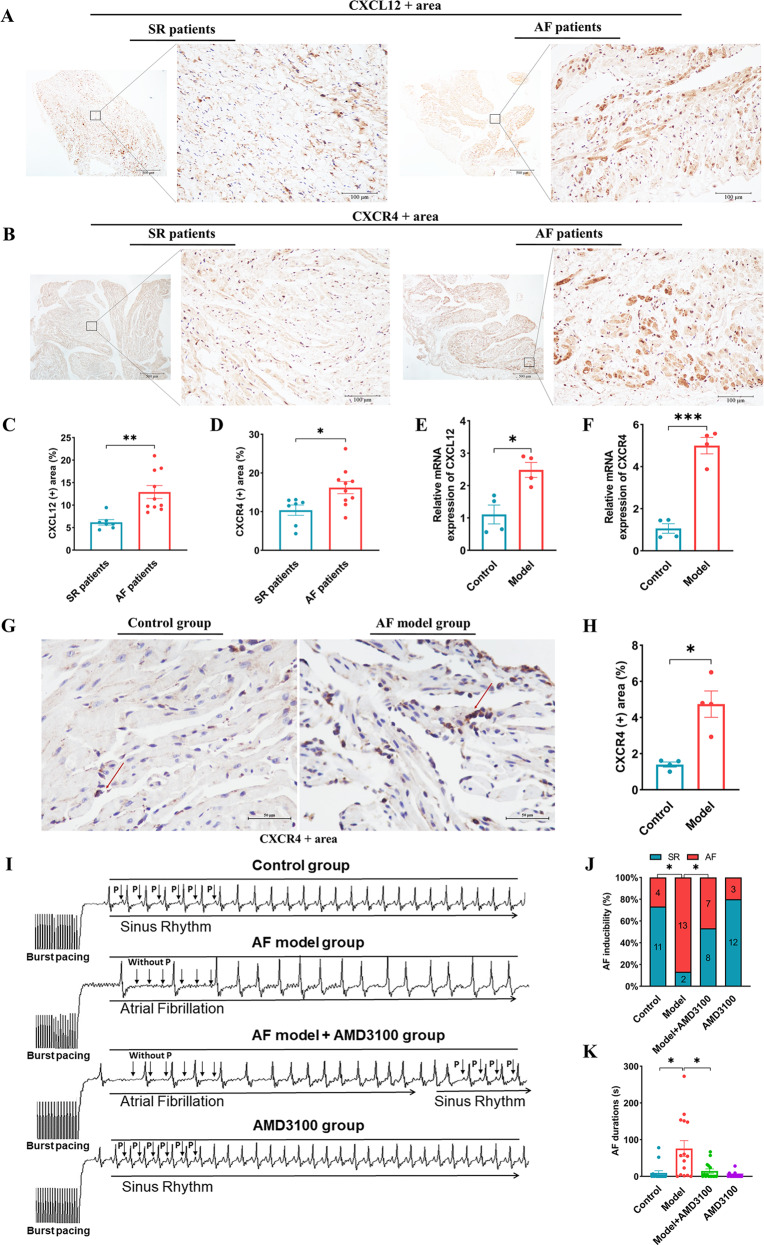
CXCL12 and CXCR4 expression in the atria of AF patients and model mice. **A**–**D** The Immunohistochemistry of atrial of control and AF patients with anti-CXCL12 and CXCR4 antibody (SR, *n* = 7; AF, *n* = 10). **E**–**F** The qPCR analysis of the mRNA levels of chemokines CXCL12 and CXCR4 in the atria of control and AF model mice (*n* = 4). **G**–**H** The Immunohistochemistry of atrial of control and AF model mice with anti-CXCR4 antibody (*n* = 4). **I**–**K** AMD3100 administration prevents the induction of AF. **I** The representative surface electrocardiogram after the transesophageal atrial burst pacing. **J** Percentage of successful AF induction (*n* = 15). **K** Duration of AF in mice after AF induction (*n* = 15). SR Sinus rhythm, AF atrial fibrillation, Scale bar: 50 μm, Results are expressed as the mean ± SEM, and n represents the number of animals in each group. *P < 0.05, **P < 0.01, ***P < 0.001.

We identified the expression level of CXCL12 and CXCR4 in AF patients and model mice. The IHC analyses demonstrated that the protein expression level of CXCL12 and CXCR4 was significantly increased in the atria of AF patients compared with controls (Fig. 3A–D). Meanwhile, the mRNA expression of CXCL12 and CXCR4 was also significantly increased in the atria of AF model mice compared with controls (Fig. 3E, F), as well as the protein expression of CXCR4 analyzed by the IHC (Fig. 3G, H). The expression validation of CXCL12 and CXCR4 was completely consistent with the results of our bioinformatics analysis, which suggested the excessively activated CXCL12/CXCR4 axis may contribute to the development of AF.

### AMD3100 administration reduces AF inducibility in AF model mice

To evaluate whether CXCL12/CXCR4 axis is involved in the development of AF, we treated AF model mice with the CXCR4 antagonist AMD3100 or vehicle for 3 weeks. The AF susceptibility and duration were evaluated by transesophageal atrial pacing. The basic ECG parameters and the cardiac electrophysiology data are presented in (Table 1). AF model mice had a longer duration of P wave, QRS wave, and SACT compared with the control group, and the duration of the P wave and SACT could be significantly decreased after the treatment of AMD3100. The AF inducibility in AF model mice was significantly higher than controls, and this phenomenon was significantly reduced with AMD3100 (Fig. 3I, J). Accordingly, the duration of AF was significantly reduced in AF model mice treated with AMD3100 compared with AF model mice treated with vehicle (Fig. 3K). There was no significant difference in AF inducibility and AF duration between the control and AMD3100 groups. These data indicate that blocking CXCL12/CXCR4 with AMD3100 reduces AF inducibility in AF model mice.

**Table 1:** Characterization of surface ECG parameters, transesophageal recording and atrial stimulation.

Parameter	Groups			
	Control (*n* = 15)	Model (*n* = 15)	Model + AMD3100 (*n* = 15)	AMD3100 (*n* = 15)
Heart rate (bpm)	393.87 ± 24.09	342.93 ± 27.59	327.13 ± 9.15	376.87 ± 23.33
P (ms)	20.74 ± 0.65^*^	25.54 ± 0.42^*#$^	21.07 ± 0.70^#^	20.20 ± 0.62^$^
PR (ms)	37.61 ± 1.45	39.72 ± 1.24	39.13 ± 1.30	37.70 ± 0.78
QRS (ms)	21.43 ± 0.40^*&^	23.66 ± 0.52^*$^	23.90 ± 0.56^&^	20.62 ± 0.36^$^
QT (ms)	65.55 ± 0.79	66.22 ± 1.09	67.24 ± 0.78	64.83 ± 0.43
SNRT (ms)	249.60 ± 12.55	276.91 ± 16.17	273.87 ± 15.48	237.87 ± 4.09
SACT (ms)	26.50 ± 3.25^*^	30.95 ± 2.79^*#$^	25.55 ± 1.53^#^	25.03 ± 1.69^$^
AVERP120 (ms)	74.00 ± 2.98	72.00 ± 3.34	75.47 ± 3.07	75.67 ± 2.38
AVERP110 (ms)	75.33 ± 3.21	74.33 ± 3.38	75.22 ± 3.00	74.34 ± 2.62
AVERP100 (ms)	72.67 ± 3.16	76.67 ± 3.54	76.84 ± 3.33	74.33 ± 2.96

*SNRT* sinus node recovery time, *SACT* sinoatrial conduction time, *AVERP* atrioventricular nodal refractory period.

All data are presented as means ± SEM, and *n* represents the number of animals in each group; *P < 0.05, Control vs. Model; ^&^*P* < 0.05, Control vs. Model + AMD3100; ^#^*P* < 0.05, Model vs. Model + AMD3100; ^$^*P* < 0.05, Model vs. AMD3100.

### AMD3100 administration inhibits atrial dilation and fibrosis

**Fig. 4:**
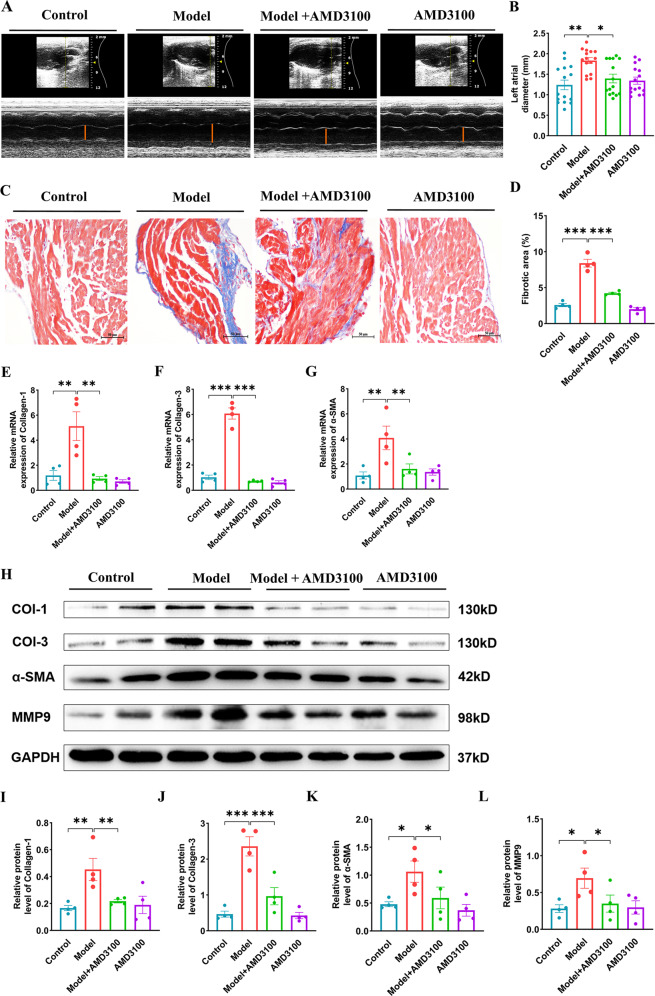
AMD3100 administration prevents atrial structural remodeling. **A**–**B** The M-mode echocardiography of the left atrial chamber (left) and the quantification of left atrial diameter (*n* = 15). **C**–**D** Collagen deposition in left atrial sections detected by Masson’s trichrome staining (left). Quantification of fibrotic areas (*n* = 4). **E–G** The qPCR analysis of the mRNA levels of α-SMA, collagen -1, and collagen-3 in the atria from four groups (*n* = 4). **H**–**L** Immunoblotting analysis of α-SMA, collagen -1, and collagen-3 and MMP9 protein levels in the atria (upper). Quantification of each protein band (lower, *n* = 4). GAPDH was used as an internal control. Scale bar: 50 μm. Results are expressed as the mean ± SEM, and n represents the number of animals in each group. *P < 0.05, **P < 0.01, ***P < 0.001.

Echocardiography indicated that the left atrial dilation observed in vehicle-treated mice of the AF model was markedly blunted in AMD3100-treated mice (Fig. 4A, B). There was no significant difference in other echocardiographic index of the left ventricular size or function (Table 2). As atrial fibrosis is the hallmark of structural remodeling in AF, we assessed whether CXCL12/CXCR4 affected the formation of atrial fibrosis. The Masson-staining showed the mice in the AF model group displayed distinct atrial fibrosis compared with the control group, which could be markedly reduced with AMD3100 treatment (Fig. 4C, D). Correspondingly, we verified the effect of AMD3100 on the mRNA and protein levels of collagen-1, collagen-3, and α-SMA in the atria of different groups. The upregulated mRNAs and proteins of collagen-1, collagen-3, and α-SMA were observed in AF model mice compared with controls. The significantly downward trend of these mRNAs and proteins were observed after the treatment of AMD3100 in AF model mice (Fig. 4E–K). Furthermore, the protein level of MMP9 was also upregulated in AF model mice, which could be markedly reduced via the treatment of AMD3100 (Fig. 4H, L). Blocking CXCL12/CXCR4 axis may ameliorate the atrial structural remodeling in AF model mice.

**Table 2:** Echocardiographic data of mice from different groups.

Parameter	Groups			
	Control (*n* = 15)	Model (*n* = 15)	Model + AMD3100 (*n* = 15)	AMD3100 (*n* = 15)
Body weight (g)	23.78 ± 0.27	23.92 ± 0.75	23.62 ± 0.55	23.82 ± 0.68
IVSd (mm)	0.82 ± 0.04	0.82 ± 0.06	0.77 ± 0.03	0.82 ± 0.04
IVSs (mm)	1.14 ± 0.07	1.19 ± 0.06	1.08 ± 0.05	1.19 ± 0.07
LVIDd (mm)	3.36 ± 0.09	3.49 ± 0.12	3.47 ± 0.08	3.42 ± 0.11
LVIDs (mm)	2.26 ± 0.13	2.35 ± 0.11	2.48 ± 0.09	2.24 ± 0.14
LVPWd (mm)	0.63 ± 0.02	0.69 ± 0.03	0.68 ± 0.02	0.71 ± 0.02
LVPWs (mm)	1.05 ± 0.06	1.00 ± 0.05	0.98 ± 0.05	1.04 ± 0.05
LVEF (%)	61.57 ± 2.88	61.77 ± 1.94	60.66 ± 2.28	65.27 ± 2.64
LVFS (%)	33.04 ± 2.30	32.65 ± 1.37	32.17 ± 1.76	35.36 ± 1.92
MV A (mm/s)	175.77 ± 13.56	228.89 ± 41.84	179.60 ± 23.95	246.27 ± 91.91
MV E (mm/s)	319.68 ± 10.97	303.55 ± 10.90	286.92 ± 17.48	290.43 ± 24.09
MV E/A ratio	1.94 ± 0.14	1.89 ± 0.24	1.79 ± 0.12	1.88 ± 0.16

*LVIDd* left ventricular internal diameter at end-diastole, *LVIDs* left ventricular internal diameter at end-systole, *LVPWd* left ventricular posterior wall thickness at end-diastole, *LVPWs* left ventricular posterior wall thickness at end-systole, *IVSd*, interventricular septal thickness at end-diastole, *IVSs*, interventricular septal thickness at end-systole, *LVEF* left ventricular ejection fraction, *LVFS* left ventricular fractional shortening, *MV E* mitral valve E wave, *MV A* mitral valve A wave.

All data are presented as means ± SEM, and n represents the number of animals in each group.

### AMD3100 administration inhibits atrial immune cells infiltration and inflammation

**Fig. 5:**
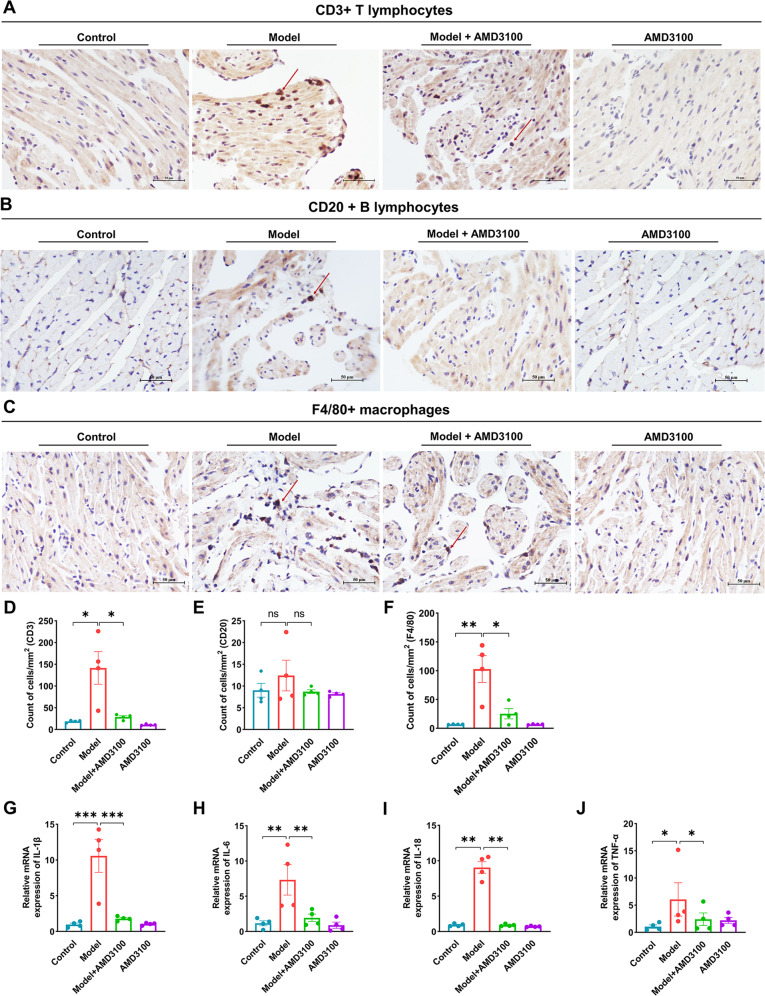
AMD3100 administration prevents the infiltration of CD3+ T lymphocytes and F4/80+ macrophages and inhibits the activation of proinflammatory. **A**, **D** Immunohistochemical staining of T lymphocytes with an anti-CD3 antibody and quantification of the CD3+ cells (*n* = 4). **B**, **E** Immunohistochemical staining of B lymphocytes with an anti-CD20 antibody and quantification of the CD20 + cells (*n* = 4). **C**, **F** Immunohistochemical staining of macrophages with an anti-F4/80 antibody and quantification of the F4/80+ cells (*n* = 4). **G**–**J** The qPCR analysis of the mRNA levels of IL-1β, IL-6, IL-18, and TNF-α in the atria from four groups (*n* = 4). Scale bar: 50 μm. Results are expressed as the mean ± SEM, and *n* represents the number of animals in each group. ns *P* > 0.05, *P < 0.05, **P < 0.01, ***P < 0.001.

To elucidate the mechanisms by which CXCR4 antagonism reduces AF inducibility, we evaluated the effect of CXCR4 on the alteration of CD3+ T lymphocytes, CD20+ B lymphocytes and F4/80+ macrophages in the atria using IHC. The infiltration of CD3+ lymphocytes in atria was markedly increased in AF model mice compared with controls, which was reduced with the treatment of AMD3100 (Fig. 5A, D). Moreover, the infiltration of F4/80+ macrophages was also significantly increased in AF model mice compared with controls. Notably, AMD3100 markedly suppressed the infiltration of F4/80+ macrophages in atria of AF model mice (Fig. 5C, F). We found the infiltration of CD20+ B lymphocytes in atria had no statistical difference between the groups (Fig. 5B, E). Meanwhile, we examined the expression of proinflammatory cytokines IL-1β, IL-6, IL-18, and TNF-α in atria. The mRNA levels of IL-1β, IL-6, IL-18, and TNF-α were higher in the atria of model mice compared with controls, while AMD3100 could markedly decrease them (Fig. 5G–J). These results suggest that blocking CXCL12/CXCR4 axis with CXCR4’s antagonist could prevent the immune cells infiltration and inflammation in AF model mice.

### AMD3100 administration inhibits CXCR4-dependent ERK1/2 and AKT/mTOR pathways

**Fig. 6:**
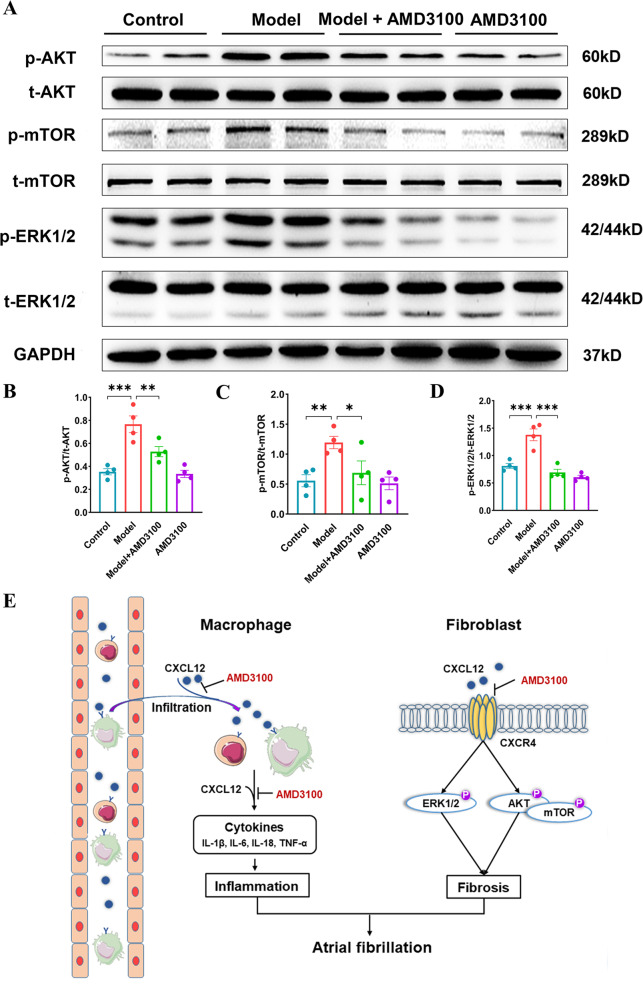
AMD3100 treatment inhibits profibrotic signaling pathways in the atria of AF model mice. **A** Immunoblotting analysis of p-AKT, AKT, p-mTOR, mTOR, p-ERK1/2, and ERK1/2 protein levels in the atria. **B**–**D** Quantification of each protein band (*n* = 4). GAPDH was used as an internal control. **E** A working model for CXCR4 mediated-AF development. AF upregulates CXCL12 expression to recruit CD3+ T lymphocytes and F4/80+ macrophages into the atrium, which trigger inflammation. Meanwhile, the upregulated CXCL12 and CXCR4 could directly activate the downstream pathways including ERK1/2 and AKT/mTOR, which induce atrial fibrosis. In contrast, pharmacological blockage of CXCR4 with AMD3100 blunted these effects. Results are expressed as the mean ± SEM, and n represents the number of animals in each group. ns *P* > 0.05, *P < 0.05, **P < 0.01, ***P < 0.001.

To elucidate the precise mechanisms by which AMD3100 inhibits AF development, we also examined the CXCR4-dependent AKT/mTOR and ERK1/2 pathways, which are important regulators of fibrogenesis. Indeed, western blot analysis observed the significant activation of AKT and mTOR in the atria of AF model mice than controls’, whereas this effect was markedly attenuated with therapy of AMD3100 (Fig. 6A–C). Moreover, the phosphorylation of ERK1/2 was more pronounced in AF model mice, which was effectively reduced after the treatment of AMD3100 (Fig. 6A, D). Taken together, these results indicate that the inhibition of CXCL12/CXCR4 axis reduces atrial structural remodeling partly by inhibiting AKT/mTOR and ERK1/2 signaling.

## Discussion

In the present study, we documented that the hyperactivation of CXCL12/CXCR4 axis was associated with the anabatic atrial inflammation and structure remodeling in AF model mice induced by Ach-CaCl_2_. Inhibiting CXCL12/CXCR4 axis with AMD3100 could significantly reduce atrial structural remodeling and AF susceptibility in AF model mice, mechanistically, via inhibiting the hyperactivation of pro-fibrosis signaling (ERK1/2 and AKT/mTOR) and inflammatory response (the recruitment of CD3+ T lymphocytes and F4/80+ macrophages). Therefore, this study suggests that the CXCL12/CXCR4 axis may be a potential therapeutic target for AF. A working model is illustrated in (Fig. 6E).

Here, we firstly identified three hub genes (CXCR4, CXCL12 and TIMP1) among the robust AF-DEGs using bioinformatics analysis, and all of them were significantly upregulated in AF patients. TIMP1 is involved in atrial extracellular matrix (ECM) remodeling and have been well demonstrated for AF’s exacerbation previously [37, 38]. The other two hub targets, CXCR4 and its endogenous ligand CXCL12, are members of the family of seven-span transmembrane GPCRs and chemokines respectively. Chemokines are a large family of small, inducible, secreted proteins that bind to coupled GPCRs on target cells and have the ability to recruit leukocytes to sites of injury, which contributes to AF development [39–42]. For example, Zhang et al. found that CXCL1/CXCR2 signaling could drive the infiltration of monocyte in atria accelerating atrial remodeling and AF after hypertension [42], while these effects could be mitigated by inhibiting CXCR2 with SB225002 (ref [41].). CXCL12/CXCR4 axis also involves in a diversity of pathological processes, such as homeostatic immune cell migration, inflammatory response, tissue repair, and cell survival, which is considered as an attractive therapeutic target both in cardiac and noncardiac diseases. Few clinical studies have reported that CXCL12 or CXCR4 is associated with AF, while their precise mechanism has not been fully understood [6, 14, 15, 43]. We demonstrated that the hyperactivation of CXCL12/CXCR4 axis in AF patients and model mice, which was consistent with the results obtained from bioinformatics analysis using five AF-related microarray datasets. Subsequently, we explored its underlying molecular mechanism in AF from the aspects of inflammatory response and fibrosis with the help of CXCR4 antagonist AMD3100 [44].

The infiltration of immune cells and related inflammatory response in atrial tissue is associated with AF [45, 46]. The infiltration of immune cells in the left atria of AF patients is mainly consist of immunologically active macrophages and a small part of CD3+ T lymphocytes [47, 48]. Macrophages can secret many inflammatory cytokines, such as TNF-α, IL-1β, IL-6, IL-18, TGF-β [49], which acts as the initiating factor of atrial structure and electric remodeling in AF [45, 50]. CD3+ T lymphocytes can also promote atrial inflammation, fibrosis, and influence the efficacy of neutrophils and macrophages [45, 48]. Although recent studies have demonstrated that CXCL12/CXCR4 axis plays a critical role in lymphocytes and macrophages’ recruitment to the sites of injury in diverse cardiovascular diseases, while its effect in AF is unknown [10, 32, 51, 52]. Here, our results demonstrated that the excessive activation of CXCL12/CXCR4 axis significantly increased the infiltration of CD3+ T lymphocytes and F4/80+ macrophages in the atria of AF model mice, while AMD3100 could markedly reduce this effect. Correspondingly, after the administration of AMD3100, the increased inflammation-associated cytokines, such as IL-1β, IL-6, IL-18, and TNF-α in the atria of AF model mice were also significantly reduced. Our findings suggest that the compensatory hyperactivation of CXCL12/CXCR4 axis in AF mice can recruit immune cells and trigger inflammation, while the antagonist of CXCR4 could significantly reduce these effects and AF susceptibility. AF itself can induce inflammation during atrial remodeling, which perpetuates the arrhythmia—the so-called ‘AF begets AF’ phenomenon. Thus, the CXCR4/CXCL12 axis may participate in the vicious cycle of “AF begets AF”. However, we did not explore the mechanism by which AF lead to the hyperactivation of CXCL12/CACR4 axis. We hypothesize that the subclinical cardiomyocyte injury caused by AF can activate the CXCL12/CXCR4 axis to promote atrial tissue repair, while the hyperactivation of CXCL12/CXCR4 axis seems to be more conducive to the formation of AF substrates, thereby contributing to AF maintenance [53, 54].

Atrial fibrosis is characterized by abnormal deposition of ECM proteins in the atria, which contribute to AF maintenance by increasing the heterogeneity of atrial conduction [37]. In this study, AMD3100 could significantly reduce atria’s excessive fibrosis in AF model mice [28, 55]. It has been demonstrated that the survival signaling pathways of ERK1/2 and AKT/mTOR are important regulators of cardiac fibrosis [55–59]. In AF model mice induced by Ach-CaCl_2_, the hyperactivation of ERK1/2 and AKT /mTOR signaling not only improve the survival of cardiomyocytes but also synchronously stimulate the proliferation and hypertrophy of and collagen production by cardiac fibroblasts aggravating atrial fibrosis and providing substrate for AF [55]. Blocking CXCL12/CXCR4 axis, an upstream regulator of ERK1/2 and AKT /mTOR signaling, with AMD3100 of CXCR4’s specific antagonist could counteract the hyperactivation of atrial fibrosis and reduce AF susceptibility [60]. Furthermore, matrix metalloproteinases (MMPs) and tissue inhibitors of matrix metalloproteinase (TIMPs) regulate ECM metabolism in the atria and participate in atrial fibrotic remodeling [61]. Although the function of MMPs in AF remains controversial, there is consistent evidence supporting that over-expressed MMP9 is associated with atrial structural remodeling and AF susceptibility, which also considered as a therapeutic target [61, 62]. Interestingly, the CXCL12/CXCR4 axis is also as an upstream regulator of MMP9 [63]. We documented that blocking CXCL12/CXCR4 axis with AMD3100 could reduce the expression of MMP9 in AF model, which may also contribute to the alleviation of atrial structural remodeling.

To the best of our knowledge, this is the first report demonstrating the hyperactivation of CXCL12/CXCR4 axis confers deterioration of AF. Nevertheless, it is unclear whether another newly discovered CXCL12 ligand, CXCR7 [64], is involved in the pathological process of AF. CXCR7 can be regulated by CXCL11 or CXCL12, which is also associated with pathological process in different diseases [30, 64]. Accordingly, further studies are deserved to explore the role of CXCR7 and its interplay with CXCR4 in AF development in the future.

## Conclusions

To summarize, this study firstly shows that hyperactivation of CXCL12/CXCR4 axis is observed in both the AF patients and AF model mice induced by Ach-CaCl_2_, associating with the increase of atrial inflammation and fibrosis. Blocking CXCL12/CXCR4 axis with ADMD3100, a specific antagonist of CXCR4, can significantly suppress atrial structural remodeling and AF susceptibility, through inhibiting pro-fibrosis signaling (ERK1/2 and AKT/mTOR) and inflammatory response (infiltration of CD3+ T lymphocytes and F4/80+ macrophages). Nevertheless, more functional experiments are warranted to provide deeper insights into the underlying mechanism and to support blocking CXCL12/CXCR4 axis as a clinical treatment strategy for AF in the future.

## Supplementary information

Supplementary file 1

Supplementary file 2

Supplementary file 3

Supplementary file 4

Supplementary file 5

Supplementary file 6

## Data Availability

All data are available upon request of the corresponding author.
