# Supplementary material for: CXCL12/CXCR4 axis as a key mediator in atrial fibrillation via bioinformatics analysis and functional identification
Source: Cell Death Dis. 2021 Aug 27;12(9):813. doi: 10.1038/s41419-021-04109-5 (PMC8397768; doi:10.1038/s41419-021-04109-5)
Supplement: Supplementary file 1 [file 41419_2021_4109_MOESM1_ESM.docx]

**Supplementary file 1:** The basic information of five microarray gene expression datasets in the GEO

| Dataset ID | Year and Country | Contributor | Number of samples | Age (years, mean ± SEM) | Male (%) | Type | GPL | Sample source |
| --- | --- | --- | --- | --- | --- | --- | --- | --- |
| GSE2240 | 2005, Germany | Barth et al.^16^ | 30 patients undergoing open heart surgery for valve repair or coronary artery bypass grafting (SR 20, AF 10) | SR 63 ± 11 vs. AF 63 ± 18 | 19 (63.3) | Microarray | GPL96 ([HG-U133A] Affymetrix Human Genome U133A Array) | Atrial appendage |
| GSE14975 | 2010 Germany | Adam et al.^17^ | 10 patients undergoing mitral valve surgery (SR 5, AF 5) | SR 67 ± 6 vs. AF 63 ± 8 | 8 (80.0) | Microarray | GPL570 ([HG-U133_Plus_2] Affymetrix Human Genome U133 Plus 2.0 Array) | Atrial appendage |
| GSE41177 | 2013 China | Yeh et al.^18^ | 19 patients underwent valvular surgery ( SR 3, AF 16） | SR 53 ± 10 vs. AF 54 ± 13 | 10 (52.6) | Microarray | GPL570 ([HG-U133_Plus_2] Affymetrix Human Genome U133 Plus 2.0 Array) | Atrial appendage |
| GSE79768 | 2016 China | Tsai et al.^19^ | 13 patients receiving surgery for mitral valve or coronary artery disease ( SR 6, AF 7) | SR 64 ± 15 vs.  AF 48 ±11 | 5 (38.5) | Microarray | GPL570 ([HG-U133_Plus_2] Affymetrix Human Genome U133 Plus 2.0 Array) | Atrial appendage |
| GSE115574 | 2019 Turkey | Deniz et al. | 30 patients receiving surgery for mitral valve ( SR 15, AF 15) | / | / | Microarray | GPL570 ([HG-U133_Plus_2] Affymetrix Human Genome U133 Plus 2.0 Array) | Atrial appendage |

Atrial fibrillation, AF; Sinus rhythm, SR; GSE, Gene Expression Omnibus Series; GPL, Gene Expression Omnibus Platform; GEO, Gene Expression Omnibus; SD, standard deviation
