# Supplementary material for: CXCL12/CXCR4 axis as a key mediator in atrial fibrillation via bioinformatics analysis and functional identification
Source: Cell Death Dis. 2021 Aug 27;12(9):813. doi: 10.1038/s41419-021-04109-5 (PMC8397768; doi:10.1038/s41419-021-04109-5)
Supplement: Supplementary file 2 [file 41419_2021_4109_MOESM2_ESM.docx]

**Supplementary file 2:** The basic information of 17 patients

| Patients | SR or AF | Age (year) | Gender | SBP  (mmHg) | DBP  (mmHg) | AOD  (mm) | LAD (mm) | RAD (mm) |
| --- | --- | --- | --- | --- | --- | --- | --- | --- |
| 1 | SR | 64 | F | 136 | 88 | 29 | 49 | 34 |
| 2 | SR | 58 | M | 108 | 72 | 32 | 45 | 36 |
| 3 | SR | 62 | M | 118 | 50 | 41 | 51 | 35 |
| 4 | SR | 45 | M | 96 | 62 | 37 | 55 | 55 |
| 5 | SR | 52 | M | 120 | 80 | 34 | 46 | 38 |
| 6 | SR | 47 | F | 110 | 80 | 25 | 42 | 25 |
| 7 | SR | 64 | F | 110 | 82 | 35 | 56 | 35 |
| 8 | AF | 60 | F | 132 | 83 | 34 | 58 | 46 |
| 9 | AF | 53 | F | 112 | 78 | 33 | 59 | 53 |
| 10 | AF | 64 | F | 136 | 88 | 29 | 49 | 34 |
| 11 | AF | 53 | M | 120 | 80 | 46 | 61 | 56 |
| 12 | AF | 51 | M | 130 | 65 | 25 | 57 | 44 |
| 13 | AF | 52 | M | 100 | 78 | 38 | 42 | 37 |
| 14 | AF | 53 | M | 112 | 67 | 32 | 67 | 41 |
| 15 | AF | 50 | F | 138 | 87 | 35 | 48 | 49 |
| 16 | AF | 64 | M | 100 | 60 | 40 | 64 | 39 |
| 17 | AF | 48 | F | 100 | 55 | 36 | 49 | 35 |

Atrial fibrillation, AF; Sinus rhythm, SR; F, Female; M, Male; LAD, Left atrial diameter; RAD, Right atrial diameter, AOD, Aorta diameter; SBP, Systolic blood pressure; DBP, Diastolic blood pressure
