# Supplementary material for: CXCL12/CXCR4 axis as a key mediator in atrial fibrillation via bioinformatics analysis and functional identification
Source: Cell Death Dis. 2021 Aug 27;12(9):813. doi: 10.1038/s41419-021-04109-5 (PMC8397768; doi:10.1038/s41419-021-04109-5)
Supplement: Supplementary file 3 [file 41419_2021_4109_MOESM3_ESM.docx]

**Supplementary file 3:** Primer sequence for RT-PCR

| **mRNA** | **Forwards** | **Rewards** |
| --- | --- | --- |
| CXCL12 | AACCCACCATGCTCATCATTC | TTTCAGGGTCATGGAGACAGTCT |
| CXCR4 | CGTCGTGCACAAGTGGATCT | GTTCAGGCAACAGTGGAAGAAG |
| Collagen-1 | GAGCGGAGAGTACTGGATCG | TACTCGAACGGGAATCCATC |
| Collagen-3 | ACGTAAGCACTGGTGGACAG | GGAGGGCCATAGCTGAACTG |
| α-SMA | GCAAACAGGAATACGACGAAGC | GCTTTGGGCAGGAATGATTTG |
| IL-1β | TGGTGTGTGACGTTCCCATT | TCGTTGCTTGGTTCTCCTTG |
| IL-6 | CTTCCATCCAGTTGCCTTCTTG | AATTAAGCCTCCGACTTGTGAAG |
| IL-18 | GCCGACTTCACTGTACAACCG | GAGGGTCACAGCCAGTCCTC |
| TNF-α | AGGGCTGTGGGACCTAAATGT | ATGGGATGAGTATGGGGCAGC |
| β-actin | ACCTCTATGCCAACACAGTG | GGACTCATCGTACTCCTGCT |
